# Early Intensive Versus Escalation Approach: Ten‐Year Impact on Disability in Relapsing Multiple Sclerosis

**DOI:** 10.1002/acn3.70131

**Published:** 2025-07-06

**Authors:** Pietro Iaffaldano, Giuseppe Lucisano, Tommaso Guerra, Francesca Caputo, Marta Simone, Massimiliano Copetti, Damiano Paolicelli, Emilio Portaccio, Francesco Patti, Paola Perini, Vincenzo Brescia Morra, Alessia Di Sapio, Matilde Inglese, Carlo Pozzilli, Giacomo Lus, Giuseppe Salemi, Erica Curti, Giovanna De Luca, Paola Valentino, Eleonora Cocco, Paola Cavalla, Carlo Avolio, Alessandra Lugaresi, Antonio Gallo, Pietro Annovazzi, Maria A. Rocca, Clara Grazia Chisari, Massimo Filippi, Maria Pia Amato, Maria Trojano, the Italian MS Register, Beatrice Biolzi, Daniele Dell’Anna, Daniele Di Giulio Cesare, Sonia Di Lemme, Chiara Di Tillio, Teresa Fonsdituri, Ilaria Maietta, Agata Marchese, Silvia Marinetto, Federica Martini, Cristiana Morano, Silvia Perugini, Giovanna Ramona Piredda, Chiara Raimondi, Ilaria Rossi, Valentina Tallarico, Stefania Treccarichi

**Affiliations:** ^1^ Department of Translational Biomedicines and Neurosciences (DiBraiN) University of Bari “Aldo Moro” Bari Italy; ^2^ CORESEARCH—Center for Outcomes Research and Clinical Epidemiology Pescara Italy; ^3^ Child Neuropsychiatry Unit, Department of Precision and Regenerative Medicine Jonic Area University of Bari “Aldo Moro” Bari Italy; ^4^ Fondazione IRCCS—“Casa Sollievo Della Sofferenza,” Unit of Biostatistics San Giovanni Rotondo Italy; ^5^ Department of NEUROFARBA University of Florence Florence Italy; ^6^ Department “GF Ingrassia,” Section of Neurosciences, Neurology Clinic University of Catania Catania Italy; ^7^ Department of Neurosciences, Multiple Sclerosis Centre‐Veneto Region (CeSMuV) University Hospital of Padua padua Italy; ^8^ Department of Neuroscience (NSRO) Multiple Sclerosis Clinical Care and Research Center, Federico II University Naples Italy; ^9^ Regional Referral MS Center, Neurological Unit Univ. Hospital San Luigi Gonzaga Orbassano Italy; ^10^ Department of Neuroscience, Rehabilitation, Ophthalmology, Genetics, Maternal and Child Health (DINOGMI) University of Genoa Genoa Italy; ^11^ IRCCS Ospedale Policlinico San Martino Genoa Italy; ^12^ Department of Human Neuroscience Sapienza University Rome Italy; ^13^ Multiple Sclerosis Center, II Division of Neurology, Department of Clinical and Experimental Medicine Second University of Naples Naples Italy; ^14^ Department of Biomedicine, Neuroscience and Advanced Diagnostics University of Palermo Palermo Italy; ^15^ Unit of Neurosciences, Department of Medicine and Surgery University of Parma Parma Italy; ^16^ Multiple Sclerosis Centre, Neurology Unit University Hospital SS Annunziata, Chieti, University G. d'Annunzio Chieti‐Pescara Italy; ^17^ Department of Medical and Surgical Sciences Institute of Neurology, Magna Graecia University Catanzaro Italy; ^18^ Department of Medical Science and Public Health, Multiple Sclerosis Center University of Cagliari Cagliari Italy; ^19^ Multiple Sclerosis Center and 1 Division of Neurology, Department of Neuroscience University of Turin & City of Health and Science University Hospital Turin Italy; ^20^ Department of Medical and Surgical Sciences University of Foggia Foggia Italy; ^21^ UOSI Riabilitazione Sclerosi Multipla IRCCS Istituto Delle Scienze Neurologiche di Bologna Bologna Italy; ^22^ Dipartimento di Scienze Biomediche e Neuromotorie Università di Bologna Bologna Italy; ^23^ Department of Advanced Medical and Surgical Sciences University of Campania “Luigi Vanvitelli” Naples Italy; ^24^ Neuroimmunology Unit, Multiple Sclerosis Center Hospital of Gallarate, ASST Della Valle Olona Gallarate (Varese) Italy; ^25^ Neuroimaging Research Unit, Division of Neuroscience IRCCS Ospedale San Raffaele Milano Italy; ^26^ Neurology Unit IRCCS Ospedale San Raffaele Milano Italy; ^27^ Vita‐Salute San Raffaele University Milano Italy; ^28^ IRCCS Fondazione Don Carlo Gnocchi Florence Italy

**Keywords:** disability trajectories, multiple sclerosis, PIRA

## Abstract

**Objective:**

To evaluate the long‐term impact of early intensive treatment (EIT) versus escalation (ESC) strategies using high‐efficacy disease‐modifying therapies (HE‐DMTs) on disability progression in relapsing multiple sclerosis (RMS).

**Methods:**

This observational study included 4878 RMS patients from the Italian Multiple Sclerosis Register. Eligible participants initiated their first disease‐modifying therapy (DMT) within 3 years of disease onset and had ≥ 5 years of follow‐up with at least three Expanded Disability Status Scale (EDSS) evaluations. Patients were categorized into the EIT group if they started with HE‐DMTs and into the ESC group if HE‐DMTs were initiated after ≥ 1 year of moderate‐efficacy therapy. Propensity score matching was performed to balance baseline characteristics. Outcomes included disability trajectories assessed using linear mixed models for repeated measures and risks of confirmed disability accrual (CDA), progression independent of relapse activity (PIRA), and relapse‐associated worsening (RAW) evaluated using Cox proportional hazards models.

**Results:**

Post‐matching analysis of 908 pairs revealed significantly slower disability progression in the EIT group compared to the ESC group. At 10 years, the delta‐EDSS difference between groups was −0.63 (95% CI: −0.83 to −0.43; *p* < 0.0001). ESC was associated with higher risks of CDA (HR 1.36, 95% CI: 1.20–1.54; *p* < 0.0001), PIRA (HR 1.22, 95% CI: 1.05–1.40; *p* = 0.0074), and RAW (HR 1.55, 95% CI: 1.17–2.05; *p* = 0.0021).

**Interpretation:**

EIT significantly reduces long‐term disability progression in RMS compared to ESC. These findings underscore the potential of EIT to optimize long‐term outcomes in RMS patients.

## 1 Introduction

Multiple sclerosis (MS) is a chronic autoimmune disease of the central nervous system, sustained by different pathological mechanisms constituting a *continuum* marked by neuroinflammation and neurodegeneration [1, 2, 3]. The accumulation of neurological disability over time, mainly driven by subclinical disease activity and progression independent of relapse activity (PIRA) events, is currently a major therapeutic challenge in MS management [4, 5].

By lowering the relapse rate and limiting disability progression, disease‐modifying treatments (DMTs) have drastically changed the treatment landscape of MS over the last few years [6]. Compared to moderately effective (ME) therapies, exposure to highly effective (HE) DMTs has been shown to be more beneficial in reducing the accumulation of impairment caused by relapse‐associated worsening (RAW) and PIRA phenomena [4, 7]. Nevertheless, the decision to start HE‐DMTs early (early intensive treatment, EIT) or reserve these therapies for escalation (ESC) after the failure of ME treatments remains a matter of debate [8]. Our capacity to accurately predict the course of MS disease is limited, making it difficult to choose the right therapy, especially for newly diagnosed individuals. To optimize long‐term outcomes and impact on disability trajectories, EIT strategies are crucial and recommended by a growing number of recent observational studies and expert opinions [9, 10].

This study aimed to examine the ten‐year impact of EIT versus ESC on disability trajectories over time and to assess the time to first 6‐months Confirmed Disability Accrual (CDA), PIRA, and RAW events, in a large cohort of relapsing MS (RMS) patients extracted from the Italian MS and related disorders (I‐MS&RD) register database [11].

## 2 Materials and Methods

### 2.1 Data Extraction

This was a study based on data extracted from the I‐MS&RD register. I‐MS&RD register was approved by the ethical committee at the “Azienda Ospedaliero—Universitaria—Policlinico of Bari” (Study REGISTRO SM001—approved on 8 July 2016) and by local ethics committees in all participating centers. Patients signed an informed consent that allows to collect and use their clinical data for research purposes. According to the Registry rules, the Scientific Committee of the I‐MS&RD register granted the approval to conduct this project and extract and use the registry data. Data extraction was executed in March 2023.

### 2.2 Study Population and Outcomes

We included in the analysis RMS patients treated with at least one DMT prescribed after 1st January 2007. In addition, a minimum period of 5 years of follow‐up after DMT initiation was required, including subjects that received their first DMT within 3 years of disease onset and had at least three Expanded Disability Status Scale (EDSS) evaluations after treatment initiation.

Patients were categorized into two groups. EIT cohort included patients who initiated treatment with HE‐DMTs, including natalizumab, alemtuzumab, ocrelizumab, cladribine, fingolimod, or mitoxantrone. Conversely, the ESC group included patients initially treated with ME‐DMTs (azathioprine, interferon‐beta products, glatiramer acetate, teriflunomide, dimethyl fumarate) and escalated to HE‐DMTs after at least 1 year.

Outcomes were defined as follows:

– Trajectories of EDSS were outlined considering the time frame from the first prescription to the last follow‐up visit and segmented in 6‐month periods, evaluating the growth of EDSS at each semester compared with the first one.
– Confirmed disability accrual (CDA) was defined as a confirmed 6‐month disability increase from study baseline, measured by EDSS (increase ≥ 1.5 points with baseline EDSS = 0; ≥ 1.0 point with baseline EDSS > 1.0, and < 5.5; ≥ 0.5 point with baseline EDSS > 6.0). Date of CDA was assigned at the first EDSS when an increase was registered. The first EDSS considered corresponds to the neurological evaluation at the initiation of the first treatment.
– RAW was defined as a CDA event in which the initial disability increase from study baseline occurred within 90 days or earlier after or 30 days or earlier before the onset of a relapse.
– PIRA was defined as a CDA event occurring more than 90 days after and more than 30 days before the onset of a relapse.

### 2.3 Statistical Analysis

In descriptive analyses, categorical data were expressed as frequency and proportion. Continuous data were expressed as mean and standard deviation (SD).

To mitigate the impact of potential biases, patients were 1:1 propensity score (PS)‐matched for characteristics at the first DMT. The quality of the procedure in each pair of matched cohorts was assessed with absolute standardized mean difference (SMD), considering SMD less than 10% acceptable, as absolute value. One‐to‐one, greedy, nearest neighbor, random matching on PS was used. A matching caliper value of 0.2 standard deviations of the PS has been used.

The following variables have been considered in the PS matching procedure: gender (male and female), age at first DMT (in years) initiation, time from disease onset to the first DMT start (in months), baseline EDSS, number of EDSS assessments from the first DMT, number of patients with relapses 2 years before DMT start, onset type (monofocal or multifocal), T2 lesion load (0, 1–2, 3–8 or ≥ 9) and gadolinium (GD)‐enhancing T1 lesion (presence/absence) before study baseline. The variable of the number of EDSS assessments from the first DMT was not included in an additional PS matching procedure performed as sensitivity analysis and included as Supporting Information.

Multiple imputation of missing data was not recommended due to the significant number of participants without data for MRI T2 lesion burden and T1 GD‐enhancing lesion; instead, a category “missing” representing subjects lacking data was considered to avoid excluding many subjects in the analysis. The follow‐up time from the first DMT start has been segmented into 6‐month periods. Therefore, if a new EDSS assessment was not completed throughout the semester period, the previous neurological evaluation was considered until the subsequent EDSS score. The disability trajectories were evaluated by applying a linear mixed model for repeated measures (LMMRM). The adjusted evolution over time of the disability accumulation was assessed by calculating the mean annual estimated EDSS changes compared to baseline estimated EDSS values (delta‐EDSS) for each treatment strategy group. LMMRM with an autoregressive correlation‐type matrix allowed evaluation of all individuals, including participants with incomplete data. The comparison between EIT and ESC was performed by evaluating the yearly differences of the delta‐EDSS (delta2‐EDSS). Cox proportional hazard regression models were applied to compare ESC vs. EIT in terms of time to first 6‐month confirmed CDA, PIRA, and RAW events. The proportional hazard (PH) assumption was ensured by testing the log of time interaction. Results of Cox regression models were expressed as hazard ratio (HR) and 95% confidence interval (95% CI) of reaching the outcomes. Kaplan–Meier (KM) curves were used to show the cumulative probabilities of reaching the outcomes. We estimated the outcomes by applying robust standard error (SE). *p* < 0.05 result in statistical significance. Analyses were performed using R 4.2.3.

## 3 Results

**FIGURE 1:**
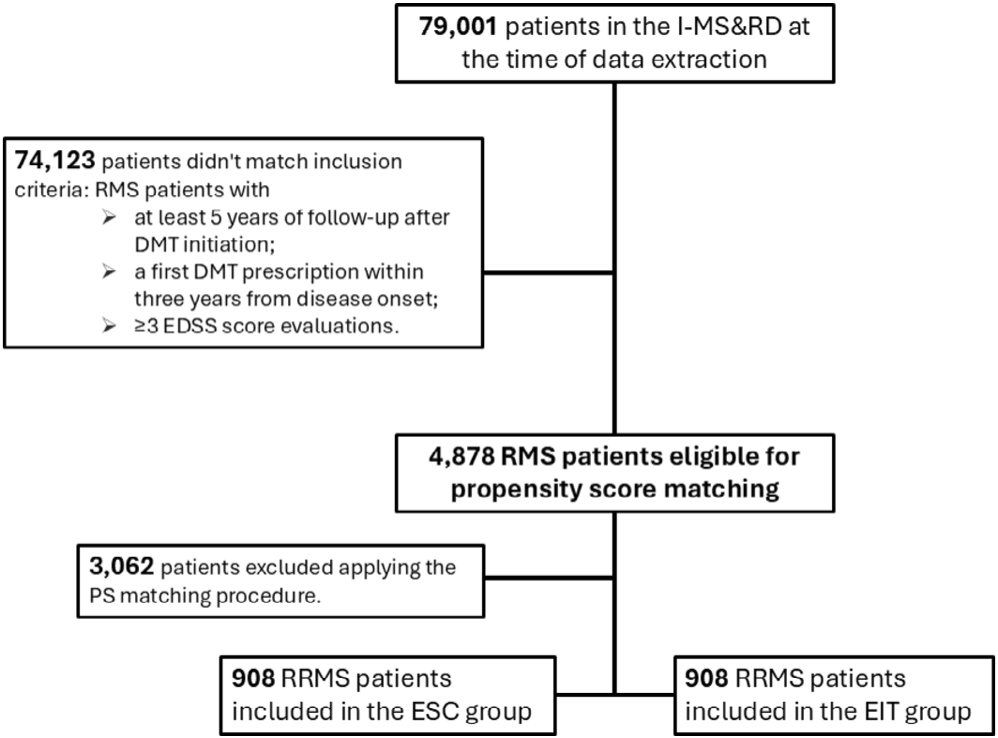
Flowchart of patients' selection procedure.

Clinical data of 79,001 patients were available in the I‐MS&RD register at the time of data extraction. After applying the inclusion criteria, we retrieved a cohort of 4878 RMS patients. Among them, 914 patients were treated with an EIT approach, while 3964 patients followed an ESC strategy. After applying the PS matching procedure, 908 patient pairs were retained for analysis, ensuring balance across baseline demographic and clinical characteristics. The flowchart of patients' selection procedure is reported in Figure 1. Before matching, significant differences were observed between the two groups: ESC patients were younger at treatment initiation (mean (SD) age: 31.15 (10.07) years in ESC vs. 32.46 (10.84) years in EIT) and had lower baseline EDSS scores (mean (SD) EDSS: 1.93 (1.30) in ESC vs. 2.71 (1.67) in EIT). After the PS matching, all variables, including EDSS, age, and lesion burden, were balanced, with SMD within the acceptable range (< 10%). Clinical and demographic baseline characteristics before and after PS matching are reported in Table 1. Treatment exposure is reported in Table 2. The median (IQR) time of exposure to ESC was 7.7 (5.3–10.5) years, with a median time of escalation to HE‐DMTs of 4.0 (2.3–6.6) years. In parallel, the median (IQR) time of exposition to HET was 5.7 (2.3–7.9) years.

**TABLE 1:** Comparison of clinical and demographic features between ESC and EIT groups before and after propensity score matching.

Variable	Before PS matching			After PS matching		
	ESC (3964)	EIT (914)	SMD	ESC (908)	EIT (908)	SMD
Female sex, *n* (%)	2633 (66.42)	608 (66.52)	0.2	593 (65.31)	604 (66.52)	2.6
Age at first DMT, mean (SD), years	31.15 (10.07)	32.46 (10.84)	12.6	32.07 (10.29)	32.40 (10.83)	3.1
Time to first DMT, mean (SD), months	11.79 (9.25)	11.52 (9.50)	−2.8	11.25 (9.08)	11.54 (9.51)	3.0
Baseline EDSS, mean (SD)	1.93 (1.30)	2.71 (1.67)	51.9	2.63 (1.60)	2.67 (1.62)	2.4
No EDSS from the first DMT, mean (SD)	19.34 (13.91)	18.22 (14.24)	−7.9	18.04 (14.05)	18.28 (14.26)	1.7
No patients with relapses 2‐years before DMT start, mean (SD)	1896 (47.83)	506 (55.36)	−15.1	498 (54.85)	502 (55.29)	−0.9
Monofocal onset, mean (SD)	3312 (83.55)	733 (80.20)	−8.7	710 (78.19)	728 (80.18)	4.9
T2 lesion load, *n* (%)						
0	57 (1.44)	23 (2.52)	7.8	22 (2.42)	23 (2.53)	0.7
1–2	84 (2.12)	17 (1.86)		16 (1.76)	16 (1.76)	
3–8	612 (15.44)	83 (9.08)		77 (8.48)	83 (9.14)	
≥ 9	640 (16.15)	196 (21.44)		193 (21.26)	194 (21.37)	
Missing	2571 (64.86)	595 (65.10)		600 (66.08)	592 (65.20)	
T1 GD + lesion, *n* (%)						
No	2121 (53.51)	513 (56.13)	5.3	509 (56.06)	509 (56.06)	0.0
Yes (at least 1)	724 (18.26)	153 (16.74)		135 (14.87)	153 (16.85)	
Missing	1119 (28.23)	248 (27.13)		264 (29.07)	246 (27.09)	

Abbreviations: DMT, disease modifying therapy; EDSS, Expanded Disability Status Scale; GD, gadolinium.

**TABLE 2:** Distribution of moderate‐efficacy DMTs before the escalation and high‐efficacy DMTs after escalation in the ESC group (A) and distribution of high‐efficacy DMTs in the EIT group (B) after PS matching.

ESC group	*n* (%)	EIT group	*n* (%)
First DMT (before the escalation)		First DMT	
Interferon ß products	655 (72.14)	Alemtuzumab	21 (2.31)
Glatiramer acetate	163 (17.95)	Fingolimod	241 (26.54)
Azathioprine	30 (3.30)	Natalizumab	498 (54.85)
Teriflunomide	15 (1.65)	Mitoxantrone	86 (9.47)
Dimethyl Fumarate	45 (4.96)	Anti‐CD20 monoclonal antibodies	24 (2.64)
		Cladribine	38 (4.19)
High‐efficacy DMTs at the escalation			
Alemtuzumab	13 (1.43)		
Fingolimod	361 (39.76)		
Natalizumab	323 (35.57)		
Mitoxantrone	10 (1.10)		
Anti‐CD20 monoclonal antibodies	131 (14.42)		
Cladribine	54 (5.95)		
Siponimod	16 (1.76)		

**FIGURE 2:**
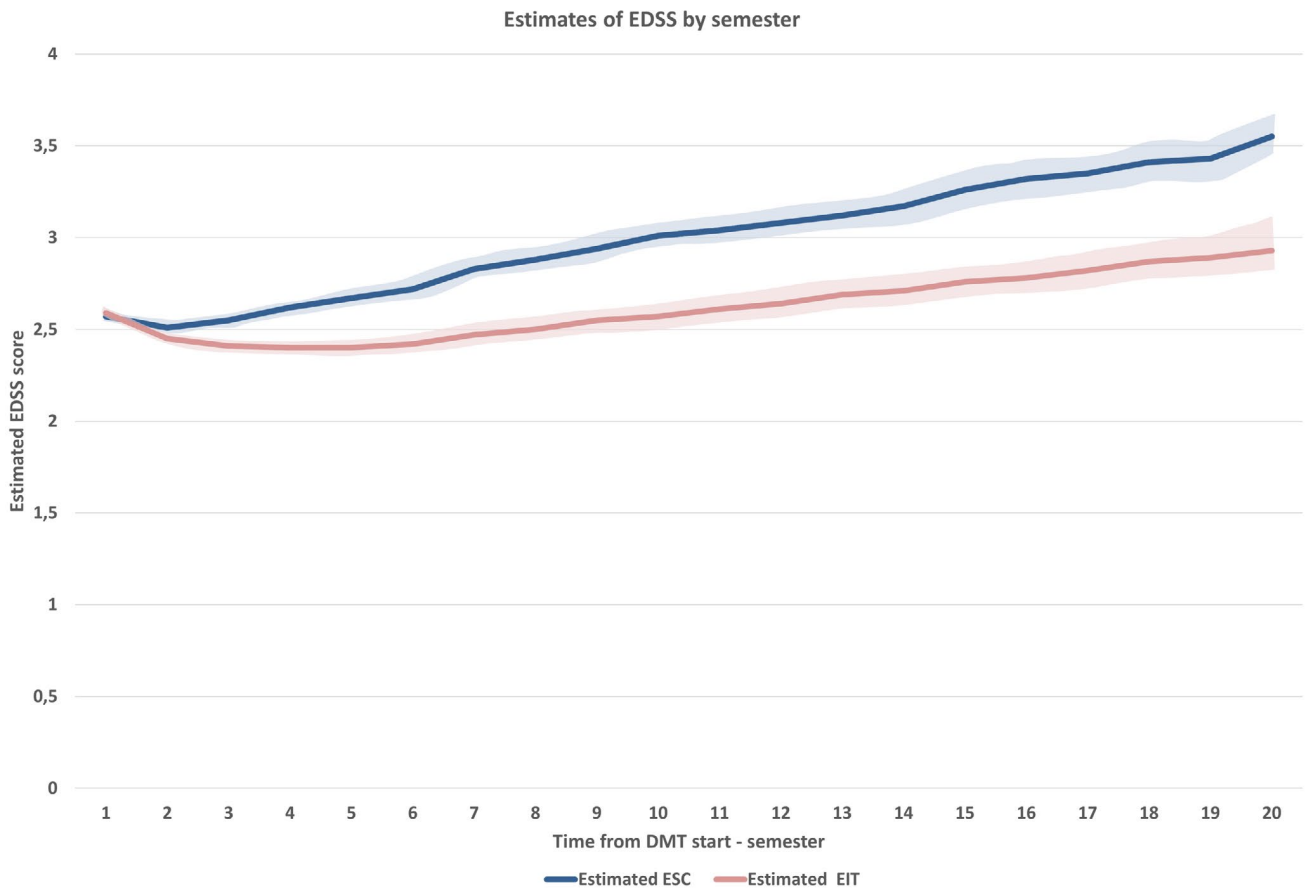
Comparison between the disability trajectories of ESC and EIT groups built through the estimated EDSS scores by semesters of follow‐up.

Longitudinal analysis of disability progression revealed significant differences between the two treatment strategies. Table 3 shows the mean estimated delta‐EDSS score differences with relative confidence intervals at each follow‐up year in the two groups compared. The estimated baseline EDSS with relative (95% confidence interval) value was 2.52 (2.33–2.71) in the ESC group and 2.45 (2.26–2.64) in the EIT group. Over the 10‐year follow‐up, mean estimated annual delta‐EDSS values were all significantly (*p* < 0.01) higher in the ESC group compared with the EIT group. At the end of the first year, the mean estimated delta‐EDSS (95% CI) difference between the groups was −0.16 (95% CI: −0.25 to −0.07; *p* = 0.0007), favoring EIT. This difference widened progressively over time, reaching −0.46 (95% CI: −0.59 to −0.33; *p* < 0.0001) at year five and −0.63 (95% CI: −0.83 to −0.43; *p* < 0.0001) at year 10. Figure 2 shows the comparison between the disability trajectories of the ESC and EIT groups built through the estimated EDSS scores by semesters.

**TABLE 3:** Estimated mean delta‐EDSS score differences between ESC and EIT groups at each follow‐up year.

Follow‐up year	Mean delta‐EDSS scores (95% CI) differences between ESC and EIT group	*p*
1 year	−0.16 (−0.25; −0.07)	0.0007
2 year	−0.24 (−0.34; −0.14)	< 0.0001
3 year	−0.31 (−0.43; −0.19)	< 0.0001
4 year	−0.40 (−0.52; −0.28)	< 0.0001
5 year	−0.46 (−0.59; −0.33)	< 0.0001
6 year	−0.45 (−0.59; −0.31)	< 0.0001
7 year	−0.48 (−0.63; −0.33)	< 0.0001
8 year	−0.56 (−0.72; −0.40)	< 0.0001
9 year	−0.56 (−0.74; −0.38)	< 0.0001
10 year	−0.63 (−0.83; −0.43)	< 0.0001

The results highlight a cumulative advantage for patients treated with EIT, which persisted and increased over the study period.

**FIGURE 3:**
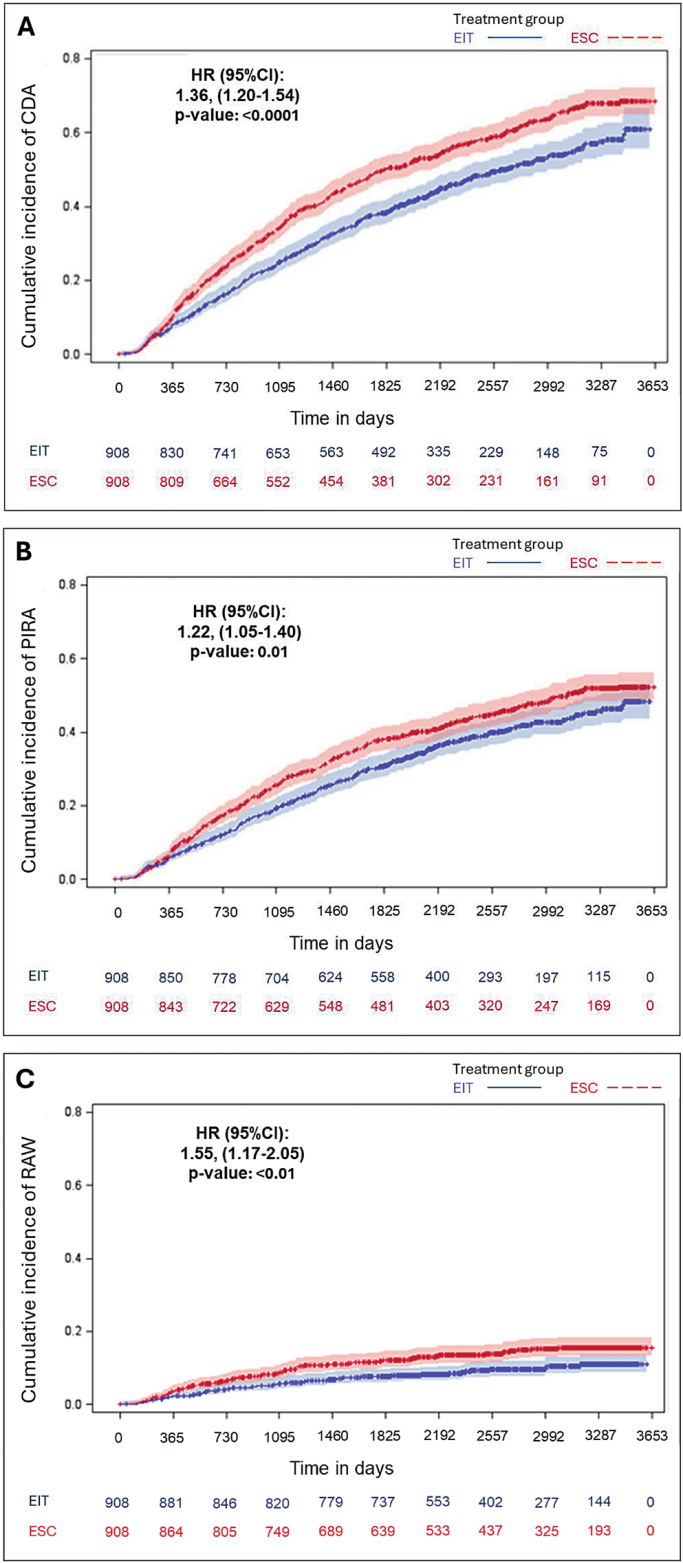
Kaplan–Meier curves for the probabilities of reaching the outcomes CDA (A), PIRA (B), and RAW (C).

The risk of CDA, PIRA, and RAW events was significantly higher in the ESC group compared to the EIT group. Cox proportional hazards models indicated that ESC was associated with a hazard ratio (HR) of 1.36 (95% CI: 1.20–1.54; *p* < 0.0001) for CDA, 1.22 (95% CI: 1.05–1.40; *p* = 0.0074) for PIRA, and 1.55 (95% CI: 1.17–2.05; *p* = 0.0021) for RAW. KM curves for the probabilities of reaching the outcomes CDA, PIRA, and RAW are shown in Figure 3. These findings underscore the effectiveness of EIT in reducing both relapse‐associated and relapse‐independent disability progression.

Additional analyses were performed, including a PS matching that did not consider the number of EDSS assessments following treatment initiation among the matching variables, resulting in a cohort of 903 patient pairs. The results were comparable, highlighting the advantage for patients treated with EIT, which persisted and increased over the study period (Supporting Information).

## 4 Discussion

In this study, we examined the effect of MS treatment strategies on 10‐year disability trajectories and the risks of CDA, PIRA, and RAW events. The results highlighted the superiority of EIT compared to ESC in the long‐term disability accrual in RMS.

One possible explanation for the initial decline of EDSS scores in the EIT group is that active RMS patients started HE‐DMTs during a high inflammatory disease activity phase, confirmed by higher EDSS scores in the Register. Following treatment initiation, EDSS scores decreased as a result of the effectiveness of prescribed HE‐DMTs. At 10 years, the delta‐EDSS difference between EIT and ESC groups was −0.63: the interpretations of our findings are consistent with prior studies demonstrating slower disability progression in EIT‐treated patients [12, 13]. Observational studies by He et al. [14], Harding et al. [15] and Spelman et al. [16] reported significant reductions in long‐term disability with EIT compared to ESC strategies. The therapeutic benefits of a tempestive treatment with HE‐DMTs were highlighted in a previous study from the I‐MS&RD register, which also evaluated long‐term disability trajectories and showed that the mean yearly delta‐EDSS values were all considerably higher for escalation approaches [17].

The novelty of our study is in evaluating and comparing the effectiveness of currently used treatment options on the risk of PIRA events, allowing us to assess in clinical practice a possible effect of current DMTs on mechanisms of silent progression. In our cohort, EIT was associated with lower risks of CDA (*p* < 0.0001), PIRA (*p* = 0.0074) and RAW (*p* = 0.0021) events.

The superiority of HE‐DMTs, compared to ME treatments, has been demonstrated in several short term randomized clinical trials (RCTs). When compared to teriflunomide, ofatumumab decreased the risk of PIRA events over a 6‐month period in ASCLEPIOS I/II [18] and similarly, ocrelizumab resulted more effective than interferon beta‐1a in preventing PIRA over a period of 12 and 24 weeks in the OPERA I/II RCT [19].

A recent study from I‐MS&RD register focused on naïve RRMS patients and demonstrated that HE‐DMTs such as ocrelizumab and natalizumab strongly suppress RAW events and have a similar impact on PIRA [20]. This is crucial, considering that patients with PIRA had significantly steeper increases in EDSS scores than those without PIRA [21]. Spelman et al. compared patients who initiated low‐to‐moderate efficacy therapy (LM‐DMT) with those who received first‐line HE‐DMT in a Swedish MS Register study and concluded that the LM‐efficacy group had a considerably higher unadjusted risk of CDW events. The HE‐DMT group also had a lower rate of PIRA, although the difference between the two groups on this measure was not statistically significant [22].

Less RAW events were found in cohorts including patients under HE‐DMTs, while PIRA events were directly related to older age, in a recent meta‐analysis [23]. RAW has less common occurrence, probably as a result of the effectiveness of therapies on neuroinflammation, but PIRA explains the majority of events leading to disability accumulation in the real‐world context, even at the earlier stages of the disease [5, 23, 24, 25].

Notwithstanding the obvious advantages of EIT, the use of HE‐DMTs in naïve treatment patients is currently restricted by obstacles such as safety profile doubts, pharmacoeconomic issues, and regulatory restrictions [8, 9, 26].

A few limitations of our study and some methodological aspects need to be taken into account while evaluating our results. The variable “number of EDSS assessments from the first DMT” was considered in the PS procedure to control differences in assessments density. PIRA and RAW depend on longitudinal EDSS data and imbalances in the frequency of assessments between groups could lead to biased estimates due to unequal opportunity for detecting disability events. In addition, patients with fewer EDSS assessments are less likely to meet the criteria for CDA, even if clinically relevant changes occurred. Acknowledging the methodological implications and limitations of using post‐treatment variable for matching procedures [27], the inclusion of this variable in the PS model helped ensure comparable observation conditions across treatment groups.

As with previous retrospective observational studies, incomplete or inaccurate data entered into the Register must be acknowledged. In the definition of CDA events, we considered solely the EDSS score and, although MRI features are considered a cornerstone in defining the disease burden, we could not include MRI data because of the lack of a systematic acquisition in the I‐MS&RD register. Acknowledging this heterogeneity, we could not include MRI data of the spinal cord in the PS matching procedure. Furthermore, in this real‐world study we are unable to account for potential confounders that could have affected results. Additionally, the generalizability of findings may vary in settings with differing healthcare policies. The study's strengths include the large, nationally representative cohort and a rigorous PS matching procedure designed to reduce bias.

In conclusion, these results from the I‐MS&RD register further confirm that an early start of HE‐DMTs reduces the risk of disability progression and improves long‐term outcomes in RMS patients compared to the ESC strategy, with a significant impact of EIT in reducing the risk of reaching a first PIRA event. Further studies are needed to establish in parallel the long‐term safety risks of the EIT approach.

## Author Contributions

P.I., G.L., T.G., and M.T.: conception and design of the study, acquisition and analysis of data, drafting the manuscript and figures; D.P., F.C., M.S., M.C., E.P., F.P., P.P., V.B.M., A.D.S., M.I., C.P., G.L., G.S., E.C., G.D.L., P.V., E.C., P.C., C.A., A.L., A.G., P.A., M.A.R., C.C.G., M.F., and M.P.A.: data acquisition and drafting a significant portion of the manuscript.

## Conflicts of Interest

The authors declare no conflicts of interest with respect to the contents of the current study, but note that the patients in the study were treated with a number of disease‐modifying drugs and that the authors have received advisory board, membership, speakers honoraria, travel support, research grants, consulting fees, or clinical trial support from the manufacturers of those drugs, including Actelion, Allergan, Almirall, Alexion, Bayer Schering, Biogen, Celgene, Excemed, Genzyme, Forward Pharma, Horizon, Ipsen, Medday, Merck Serono, Mylan, Novartis, Sanofi, Roche, Teva, and their local affiliates.

## Supporting information


Data S1.

## Data Availability

Anonymized data will be shared on reasonable request from a qualified investigator.
