# Supplementary material for: Early Intensive Versus Escalation Approach: Ten‐Year Impact on Disability in Relapsing Multiple Sclerosis
Source: Ann Clin Transl Neurol. 2025 Jul 6;12(10):2012–9. doi: 10.1002/acn3.70131 (PMC12516235; doi:10.1002/acn3.70131)

**Supplementary material**

**Figure 1.** Flowchart of patients’ selection procedure (PS matching procedure not including the number of EDSS evaluations after the first treatment).

**.
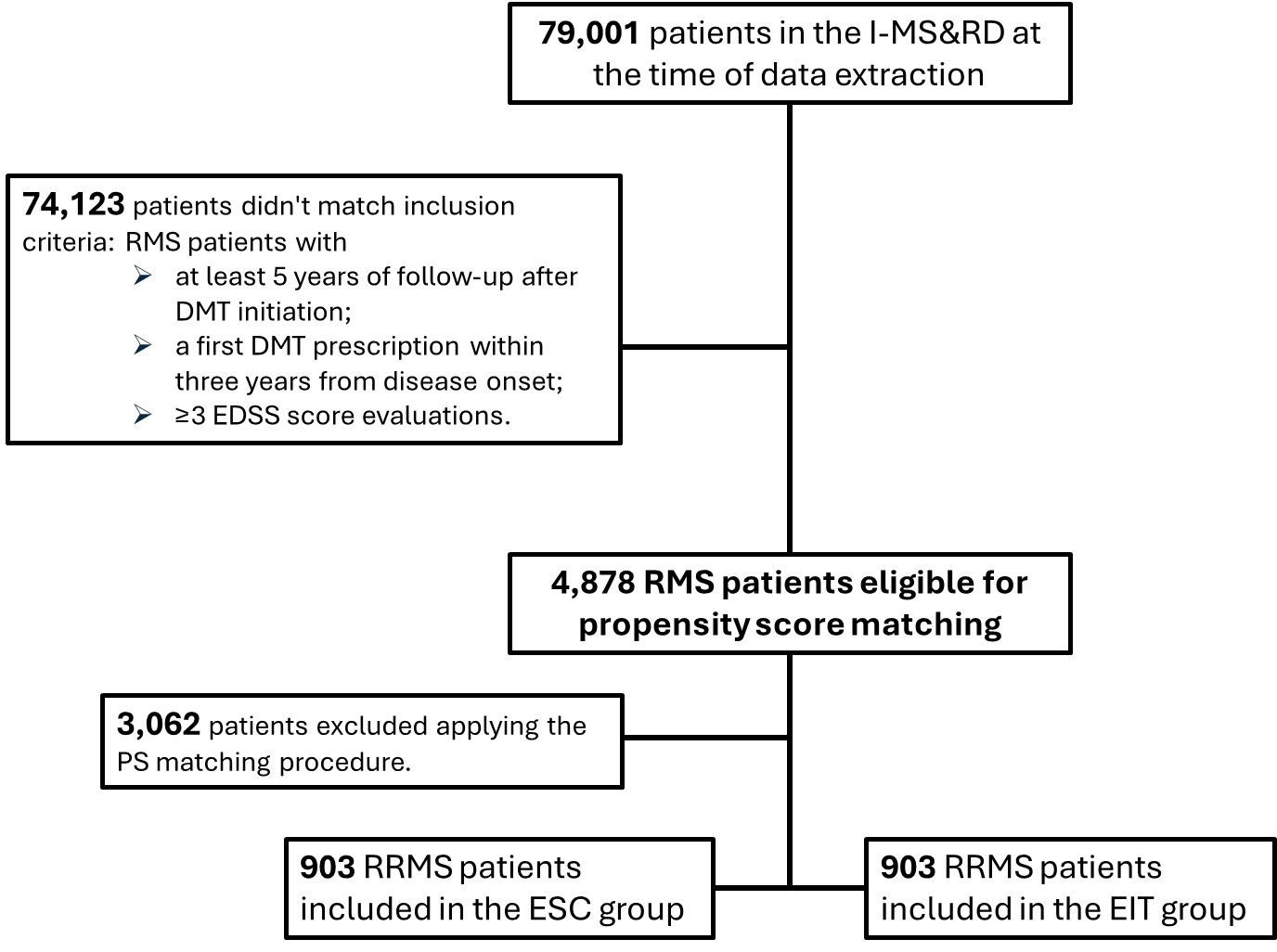
**

**Table 1.** Comparison of clinical and demographic features between ESC and EIT groups before and after propensity score matching.

|  | **Before PS matching** | | | **After PS matching** | | |
| --- | --- | --- | --- | --- | --- | --- |
| **VARIABLE** | **ESC (3964)** | **EIT (914)** | **SMD** | **ESC (903)** | **EIT (903)** | **SMD** |
| **Female sex, n (%)** | 1331 (33.58) | 306 (33.48) | 0.2 | 600 (66.45) | 599 (66.33) | -0.2 |
| **Age at first DMT, mean (SD), years** | 31.15 (10.07) | 32.46 (10.84) | 12.6 | 32.52 (10.44) | 32.37 (10.84) | -1.4 |
| **Time to first DMT, mean (SD), months** | 11.79 (9.25) | 11.52 (9.50) | -2.8 | 11.16 (8.69) | 11.57 (9.53) | 4.5 |
| **Baseline EDSS, mean (SD)** | 1.93 (1.30) | 2.71 (1.67) | 51.9 | 2.64 (1.60) | 2.64 (1.60) | 0.2 |
| **No patients with relapses 2-years before DMT start, mean (SD)** | 1896 (47.83) | 506 (55.36) | -15.1 | 475 (52.60) | 496 (54.93) | -4.7 |
| **Monofocal onset, mean (SD)** | 3312 (83.55) | 733 (80.20) | -8.7 | 705 (78.07) | 725 (80.29) | 5.5 |
| **T2 lesion load, n (%)** |  |  |  |  |  |  |
| 0 | 57 (1.44) | 23 (2.52) | 7.8 | 21 (2.33) | 23 (2.55) | 1.4 |
| 1-2 | 84 (2.12) | 17 (1.86) |  | 15 (1.66) | 15 (1.66) |  |
| 3-8 | 612 (15.44) | 83 (9.08) |  | 74 (8.19) | 83 (9.19) |  |
| ≥9 | 640 (16.15) | 196 (21.44) |  | 180 (19.93) | 193 (21.37) |  |
| Missing | 2571 (64.86) | 595 (65.10) |  | 613 (67.88) | 589 (65.23) |  |
| **T1 GD + lesion, n (%)** |  |  |  |  |  |  |
| No | 2121 (53.51) | 513 (56.13) | 5.3 | 498 (55.15) | 508 (56.26) | 2.2 |
| Yes (at least 1) | 724 (18.26) | 153 (16.74) |  | 151 (16.72) | 150 (16.61) |  |
| Missing | 1119 (28.23) | 248 (27.13) |  | 254 (28.13) | 245 (27.13) |  |

Abbreviations: DMT, disease modifying therapy; EDSS, Expanded Disability Status Scale; GD, gadolinium.

**Table 2.** Distribution of moderate-efficacy DMTs before the escalation and high-efficacy DMTs after escalation in the ESC group (A) and distribution of high-efficacy DMT in the EIT group (B) after PS matching.

1. **ESC group**

| **First DMT (before the escalation)** | n (%) |
| --- | --- |
| Interferon ß products | 637 (70.54) |
| Glatiramer acetate | 155 (17.17) |
| Azathioprine | 38 (4.21) |
| Teriflunomide | 16 (1.77) |
| Dimethyl Fumarate | 57 (6.31) |
| **High-efficacy DMTs at the escalation** |  |
| Alemtuzumab | 13 (1.44) |
| Fingolimod | 348 (38.54) |
| Natalizumab | 335 (37.10) |
| Mitoxantrone | 11 (1.22) |
| Anti-CD20 | 129 (14.28) |
| Cladribine | 52 (5.76) |
| Siponimod | 15 (1.66) |

1. **EIT group**

| **First DMT** | **n (%)** |
| --- | --- |
| Alemtuzumab | 21 (2.33) |
| Fingolimod | 242 (26.80) |
| Natalizumab | 494 (54.71) |
| Mitoxantrone | 84 (9.30) |
| Anti-CD20 | 24 (2.65) |
| Cladribine | 38 (4.21) |

**Table 3.** Estimated mean delta-EDSS score differences between ESC and EIT groups at each follow-up year.

| **Follow-up year** | **Mean delta-EDSS scores (95% CI) differences between ESC and EIT group** | **p-value** |
| --- | --- | --- |
| 1 year | -0.13 (-0.20;-0.06) | 0.0007 |
| 2 year | -0.19 (-0.27;-0.11) | <.0001 |
| 3 year | -0.21 (-0.31;-0.11) | <.0001 |
| 4 year | -0.30 (-0.41;-0.19) | <.0001 |
| 5 year | -0.41 (-0.53;-0.29) | <.0001 |
| 6 year | -0.40 (-0.53;-0.27) | <.0001 |
| 7 year | -0.43 (-0.57;-0.29) | <.0001 |
| 8 year | -0.49 (-0.65;-0.33) | <.0001 |
| 9 year | -0.48 (-0.67;-0.29) | <.0001 |
| 10 year | -0.56 (-0.77;-0.35) | <.0001 |

**Figure 2.** Kaplan-Meier curves for probabilities of reaching the outcomes CDA (A), PIRA (B) and RAW (C).


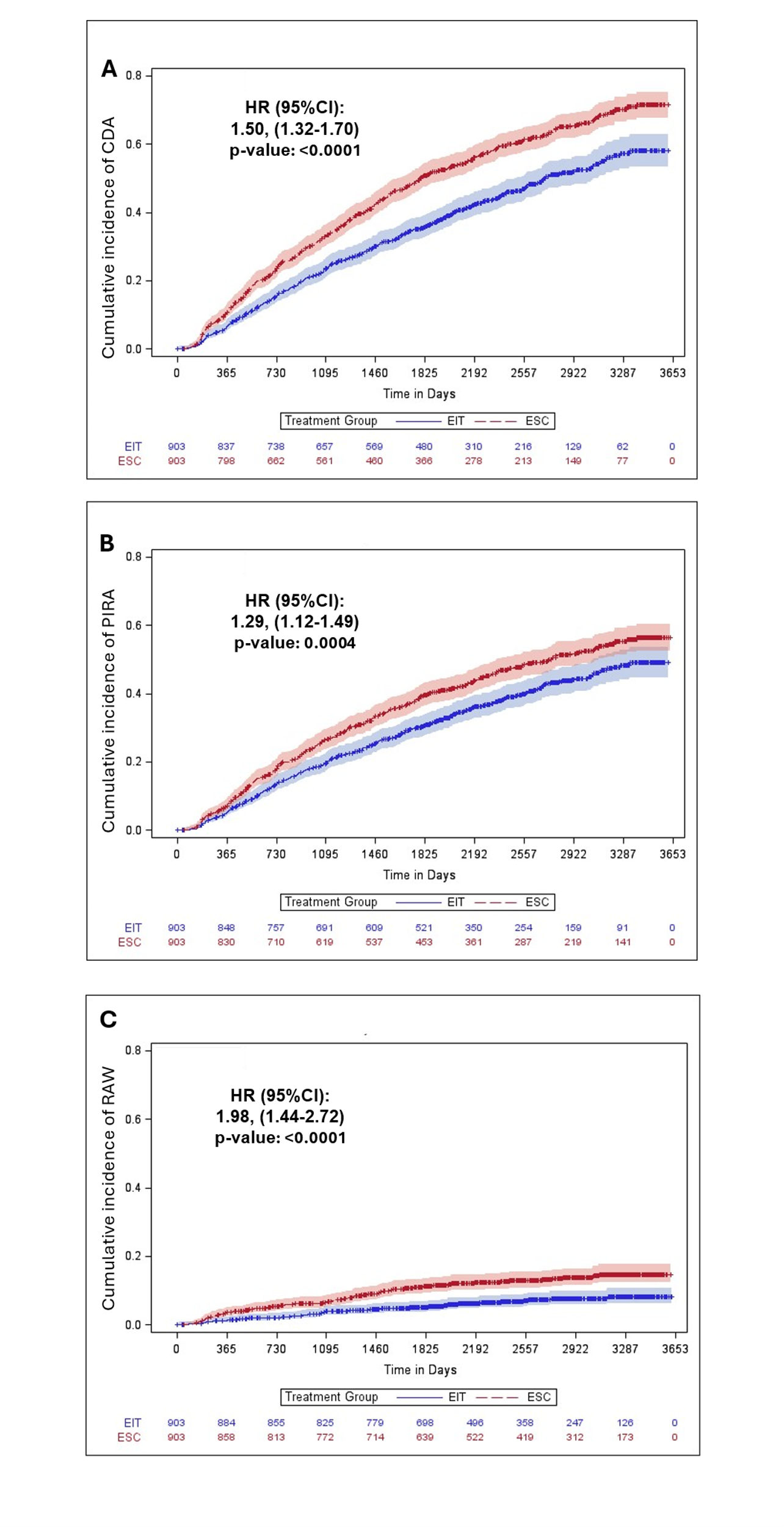

Supplement: Supplementary file 1 — Data S1. [file ACN3-12-2012-s001.docx]
